# Supplementary material for: Conditional chemoconnectomics (cCCTomics) as a strategy for efficient and conditional targeting of chemical transmission
Source: eLife. 2024 Apr 30;12:RP91927. doi: 10.7554/eLife.91927 (PMC11060718; doi:10.7554/eLife.91927)
Supplement: Figure 3—source data 3. [file elife-91927-fig3-data3.zip › Figure 3 source data3 part 2.zip!/Figure 3-Source data 3-part2/1052-13-CRISPResso_Report_ejedac2.zip!/CRISPResso_on_1052_14/3b.Insertion_deletion_substitutions_size_hist.pdf]

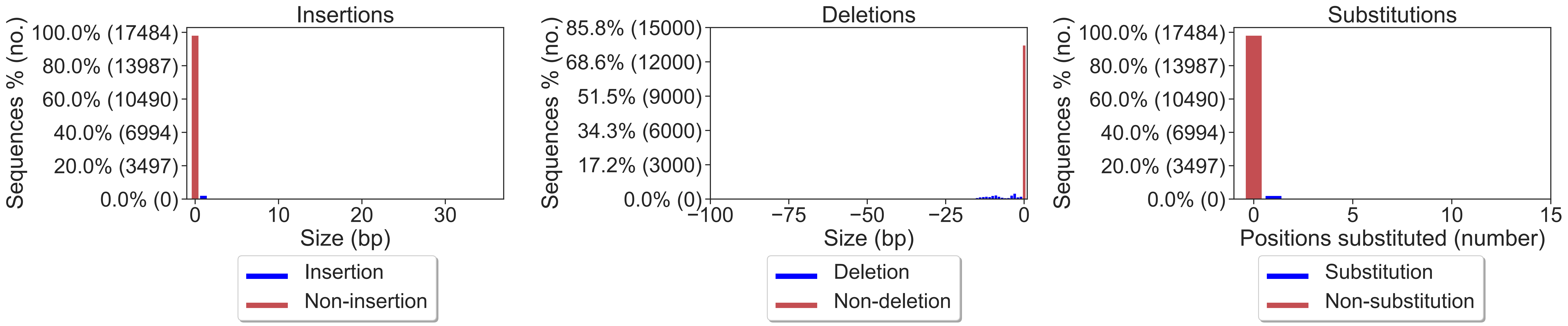

Insertions
Sequences % (no.)
100.0% (17484)
80.0% (13987)
60.0% (10490)
40.0% (6994)
20.0% (3497)
0.0% (0)
0
10
20
30
Size (bp)
Insertion
Non-insertion
Deletions
85.8% (15000)
68.6% (12000)
51.5% (9000)
34.3% (6000)
17.2% (3000)
0.0% (0)
−100
−75
−50
−25
0
Deletion
Non-deletion
Substitutions
5
15
Positions substituted (number)
Substitution
Non-substitution
